# Smoking accelerates renal cystic disease and worsens cardiac phenotype in *Pkd1*-deficient mice

**DOI:** 10.1038/s41598-021-93633-7

**Published:** 2021-07-14

**Authors:** Marciana V. Sousa, Andressa G. Amaral, Jessica A. Freitas, Gilson M. Murata, Elieser H. Watanabe, Bruno E. Balbo, Marcelo D. Tavares, Renato A. Hortegal, Camila Rocon, Leandro E. Souza, Maria C. Irigoyen, Vera M. Salemi, Luiz F. Onuchic

**Affiliations:** 1 Divisions of Nephrology and Molecular Medicine, Department of Medicine, University of São Paulo School of Medicine, Avenida Dr. Arnaldo, 455 - Sala 4304, São Paulo, SP 01246-903 Brazil; 2 Heart Institute, University of São Paulo School of Medicine, São Paulo, Brazil

**Keywords:** Genetics, Molecular biology, Cardiology, Diseases, Nephrology

## Abstract

Smoking has been associated with renal disease progression in ADPKD but the underlying deleterious mechanisms and whether it specifically worsens the cardiac phenotype remain unknown. To investigate these matters, *Pkd1*-deficient cystic mice and noncystic littermates were exposed to smoking from conception to 18 weeks of age and, along with nonexposed controls, were analyzed at 13–18 weeks. Renal cystic index and cyst-lining cell proliferation were higher in cystic mice exposed to smoking than nonexposed cystic animals. Smoking increased serum urea nitrogen in cystic and noncystic mice and independently enhanced tubular cell proliferation and apoptosis. Smoking also increased renal fibrosis, however this effect was much higher in cystic than in noncystic animals. *Pkd1* deficiency and smoking showed independent and additive effects on reducing renal levels of glutathione. Systolic function and several cardiac structural parameters were also negatively affected by smoking and the *Pkd1*-deficient status, following independent and additive patterns. Smoking did not increase, however, cardiac apoptosis or fibrosis in cystic and noncystic mice. Notably, smoking promoted a much higher reduction in body weight in *Pkd1*-deficient than in noncystic animals. Our findings show that smoking aggravated the renal and cardiac phenotypes of *Pkd1*-deficient cystic mice, suggesting that similar effects may occur in human ADPKD.

## Introduction

Autosomal dominant polycystic kidney disease (ADPKD) is the most common life-threatening monogenic human disease, with estimated prevalences of 1:543 to 1:4000 in different populations^1^. This disease is responsible for 5–10% of patients who reach end-stage kidney disease (ESKD), its main medical burden^1^. Mutations in *PKD1* (*polycystic kidney disease 1*) account for 64–85% of cases whereas almost all remaining ones are caused by mutations in *PKD2* (*polycystic kidney disease 2*)^2,3^. Mutations in *GANAB* (*glucosidase II α subunit*) and *DNAJB11* (*DnaJ homolog subfamily B member 11*) were identified in an absolute minority of cases^4,5^. Although the kidney phenotype is predominant, ADPKD is a systemic disorder, comprising a number of extrarenal manifestations such as liver cysts, intracranial aneurysm and cardiac valve and myocardial abnormalities^6,7^.

Clinical studies have reported accelerating effects of environmental agents on kidney disease in ADPKD, including high salt intake^8^ and smoking^9,10^. The mechanisms involved in such described effects, however, remain unclear. A recent report, on the other hand, found that smoking status did not influence renal survival in this disease^11^. Similarly, in vitro and in vivo experimental data suggested a detrimental effect for caffeine^12,13^, which was not confirmed by recent clinical studies^14,15^.

Smoking was found to be a risk factor of progression to ESKD, at least in men, in patients with chronic kidney disease (CKD)^16^. Its potential detrimental actions on the kidneys include endothelial lesion^16^; inflammatory and pro-proliferative effects; redox imbalance^17^; albuminuria^18^; decrease in renal plasma flow and glomerular filtration rate^19,20^; and increase in sympathetic activity and renal vascular resistance^19,20^. The specific effects of smoking on the ADPKD kidney, however, are unclear. Given the nature of tobacco adverse consequences and the properties of polycystins 1 and 2, the *PKD1* and *PKD2* products, it is unknown whether significant effects of smoking on ADPKD are disease-specific, in addition to common effects on other nephropathies. Specific effects could potentially include acceleration of cyst growth, cystogenesis, renal fibrosis and/or hemodynamic dysfunction.

Smoking is also associated with increased risk of cardiovascular disease in CKD and arterial calcification in ESKD patients^21^. Several studies revealed distinct aspects of cardiac structural alterations and/or dysfunction in *Pkd1* and *Pkd2*-deficient mouse models^22–27^. The relevance of such data is supported by the observed association between ADPKD and idiopathic dilated cardiomyopathy (IDCM)^7^. It is unknown, however, whether smoking has specific detrimental effects on the ADPKD heart, in addition to its known deleterious cardiac consequences.

Elucidation and better understanding of this unclear smoking-ADPKD scenario, which includes previously established association between smoking and renal disease progression, recent controversial data on this issue, nonevaluated potential effects of smoking on cardiac phenotype and lack of information on underlying mechanisms, required appropriate investigation using an animal model orthologous to ADPKD. In this context, we analyzed the effects of chronic exposure to tobacco on renal and cardiac phenotypes in a *Pkd1*-deficient cystic mouse, phenotypically similar to human ADPKD. This study revealed that smoking aggravated the renal and cardiac phenotypes, suggesting that similar effects are also likely to occur in human ADPKD.

## Results

### Analyzed cystic mice included *Pkd1*^flox/flox^:*Nestin*^cre^ and *Pkd1*^flox/-^:*Nestin*^cre^ animals

After the establishment of our experimental groups, a surveillance protocol of genotype control identified that *Nestin*^cre^-mediated *Pkd1* inactivation had gone germline in the colony, leading to the generation of some noncystic *Pkd1*^flox/-^ and some cystic *Pkd1*^flox/-^:*Nestin*^cre^ mice. At this point we realized that the *Pkd1*^flox/-^:*Nestin*^cre^ mouse consists in a model closer to human ADPKD1 than *Pkd1*^flox/flox^:*Nestin*^cre^, given its *Pkd1*-haploinsufficiency background. In this scenario, we decided to assemble our experimental cystic groups with statistically balanced numbers of *Pkd1*^flox/flox^:*Nestin*^cre^ and *Pkd1*^flox/-^:*Nestin*^cre^ animals (CY mice), representing in a balanced way the best and the classical cystic models. The procedures and statistical analyses performed to guarantee appropriately balanced groups are described in “Methods”. In the only analysis for which we could not exclude significant bias, we used the two genotypes as a fixed factor in the multifactorial analysis. Noncystic *Pkd1*^flox/-^ mice, however, were excluded from the study, to avoid the potential interference of *Pkd1* haploinsufficiency in the noncystic groups. Noncystic animals, therefore, were all *Pkd1*^flox/flox^. In this context, the groups included cystic mice exposed to smoking (CYS), noncystic animals exposed to smoking (NCS), cystic nonsmokers (CY) and noncystic nonsmokers (NC).

### A high proportion of renal cysts are derived from collecting ducts/distal tubules in CY mice

**Figure 1:**
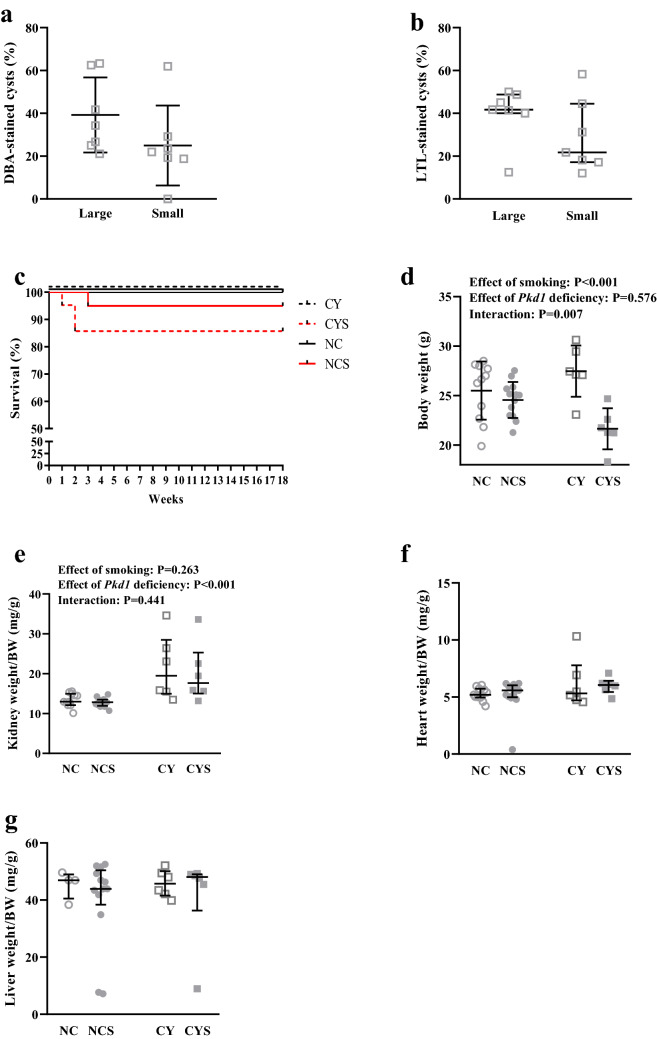
Proportion of DBA-stained (**a**) and LTL-stained (**b**) large and small renal cysts in CY mice at 18 weeks of life. (**c**) Comparative analysis of survival among CY (n = 14), CYS (n = 21), NC (n = 20) and NCS (n = 20) mice. (**d**) Analysis of the effects of smoking and *Pkd1* deficiency in body weight at 16 weeks on NC (n = 11), NCS (n = 13), CY (n = 6) and CYS (n = 6) animals. (**e**) Analysis of the effects of smoking and *Pkd1* deficiency in renal weight/body weight at 16 weeks on NC (n = 11), NCS (n = 13), CY (n = 6) and CYS (n = 6) mice. (**f**) Analysis of the effects of smoking and *Pkd1* deficiency in heart weight/body weight ratio on the four experimental groups. (**g**) Analysis of the effects of smoking and *Pkd1* deficiency in liver weight/body weight on the four experimental groups. These analyses were performed using unpaired t-test (**a**), Mann–Whitney U test (**b**), log-rank test (**c**), two-way analysis of variance (**d**) and aligned rank transform followed by two-way analysis of variance (**e**–**g**).

To evaluate the origin of the renal cysts in CY mice at 18 weeks of life, we analyzed the *Dolichos biflorus* agglutinin (DBA, a collecting duct/distal tubule marker) and *Lotus tetragonolobus* lectin (LTL, a proximal tubule marker) staining patterns in the renal cysts. DBA positive cysts accounted for 39.2 ± 17.5% of large cysts and 25.0 ± 18.7% of small cysts (Fig. 1a). When positive, LTL staining was virtually always observed only in a fraction of the cyst-lining cells, including 41.7% (40.0–48.8) of large cysts and 21.8% (17.2–44.5) of small cysts (Fig. 1b). Representative images are shown in Supplementary Figures S1a and S1b.

### Smoking does not affect fertility and survival in cystic and noncystic mice

Noncystic *Pkd1*^flox/flox^ and cystic *Pkd1*^flox/flox^:*Nestin*^cre^/*Pkd1*^flox/-^:*Nestin*^cre^ mice were exposed to smoking from conception to 18 weeks of age. Fundamental parameters were analyzed to confirm the experimental protocol viability. Pregnant females averaged seven pups per litter, the average litter size expected for C57BL/6 isogenic mice^28^. This observation indicated no significant embryonic lethality. The observed genotype frequencies also followed the Mendelian pattern of inheritance, with 51% of the offsprings displaying the Cre recombinase transgene and 49% lacking it. These data demonstrated that our smoking protocol did not exert deleterious selective pressure on cystic animals during embryonic development. Similarly, exposure to smoking did not impact on the offspring sex ratio. Weaning was performed at 5 weeks due to reduced growth in smoking animals.

Given the required population size, survival was the only analysis performed with groups not statistically balanced for the genotypes. Assessed at 18 weeks, no deaths were observed in nonsmoking mice, however no significant differences in survival were detected among NC, NCS, CY and CYS animals. A trend of decreased survival, however, was observed in CYS mice compared to NC animals (P = 0.083; Fig. 1c).

### Exposure to smoking substantially reduces body weight in cystic animals

Smoking significantly reduced body weight (BW) (P < 0.001) while the *Pkd1*-deficient cystic phenotype per se did not. Interestingly, our analysis revealed positive interaction between the *Pkd1*-deficient phenotype and smoking (P = 0.007), since the reduction in BW induced by smoking was significantly higher in cystic than in noncystic mice (27.5 ± 2.6 to 21.6 ± 2.1 g *versus* 25.5 ± 2.9 to 24.6 ± 1.8 g; Fig. 1d).

Cystic animals displayed a higher kidney weight (KW)/BW ratio than noncystic animals at 18 weeks (P < 0.001) but exposure to smoking did not impact it [12.84 mg/g (11.84–14.74) in NC, 13.10 mg/g (12.05–13.70) in NCS, 19.36 mg/g (15.13–30.09) in CY, and 17.46 mg/g (14.67–25.42) in CYS; Fig. 1e]. Heart weight (HW)/BW and liver weight (LW)/BW did not differ among the NC, NCS, CY and CYS groups (Fig. 1f,g).

### Smoking worsens renal function in cystic and noncystic mice

**Figure 2:**
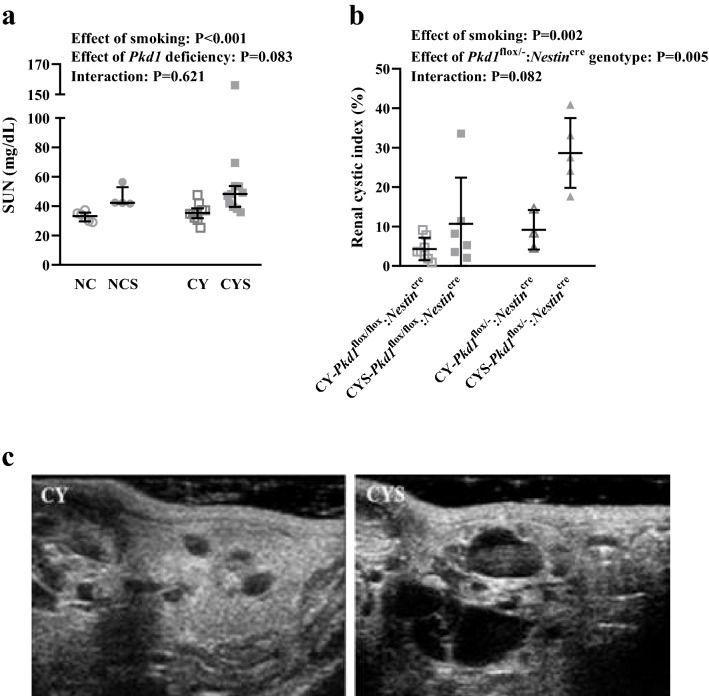
(**a**) Analysis of the effects of smoking and *Pkd1* deficiency in SUN on NC (n = 6), NCS (n = 4), CY (n = 10) and CYS (n = 11) mice. (**b**) Analysis of the effects of smoking and the *Pkd1*^flox/-^:*Nestin*^cre^ genotype in the global renal cystic index on CY-*Pkd1*^flox/flox^:*Nestin*^cre^ (n = 8), CYS-*Pkd1*^flox/flox^:*Nestin*^cre^ (n = 6), CY-*Pkd1*^flox/-^:*Nestin*^cre^ (n = 3) and CYS-*Pkd1*^flox/-^:*Nestin*^cre^ (n = 5) mice. These analyses were performed using aligned rank transform followed by two-way analysis of variance. (**c**) Representative images of the renal cystic phenotype in the absence and presence of smoking.

Smoking led to significant increase in serum urea nitrogen (SUN) in cystic and noncystic animals (P < 0.001). It must be noted that the cystic phenotype per se does not significantly affect SUN at the evaluated age [33.14 mg/dL (29.58–35.61) in NC, 42.24 mg/dL (41.81–52.90) in NCS, 35.33 mg/dL (31.69–38.42) in CY, and 48.24 mg/dL (39.47–53.72) in CYS; Fig. 2a].

### Smoking aggravates renal cystic disease

Renal ultrasonography was performed at 16 weeks of age. Since the calculated maximum 95% CI effect was > 0.1, the *Pkd1*^flox/flox^:*Nestin*^cre^ or *Pkd1*^flox/-^:*Nestin*^cre^ genotypes were applied as a fixed factor in the 2-way ANOVA. This analysis revealed an independent effect of smoking on the cystic index (P = 0.002), an effect also verified for the *Pkd1*^flox/-^:*Nestin*^cre^ genotype (P = 0.005) (Fig. 2b). Our data also showed a nonsignificant trend of positive interaction between smoking and the *Pkd1*^flox/-^:*Nestin*^cre^ genotype, expressed as a higher numerical increase in cystic index induced by smoking in *Pkd1*^flox/-^:*Nestin*^cre^ than in *Pkd1*^flox/flox^:*Nestin*^cre^ mice (9.19 ± 5.01% to 28.65 ± 8.84% *versus* 4.29 ± 2.85% to 10.68﻿ ± 11.70%; Fig. 2b). Representative images are shown in Fig. 2c.

### Exposure to smoking affects the cardiac phenotype in cystic animals

**Figure 3:**
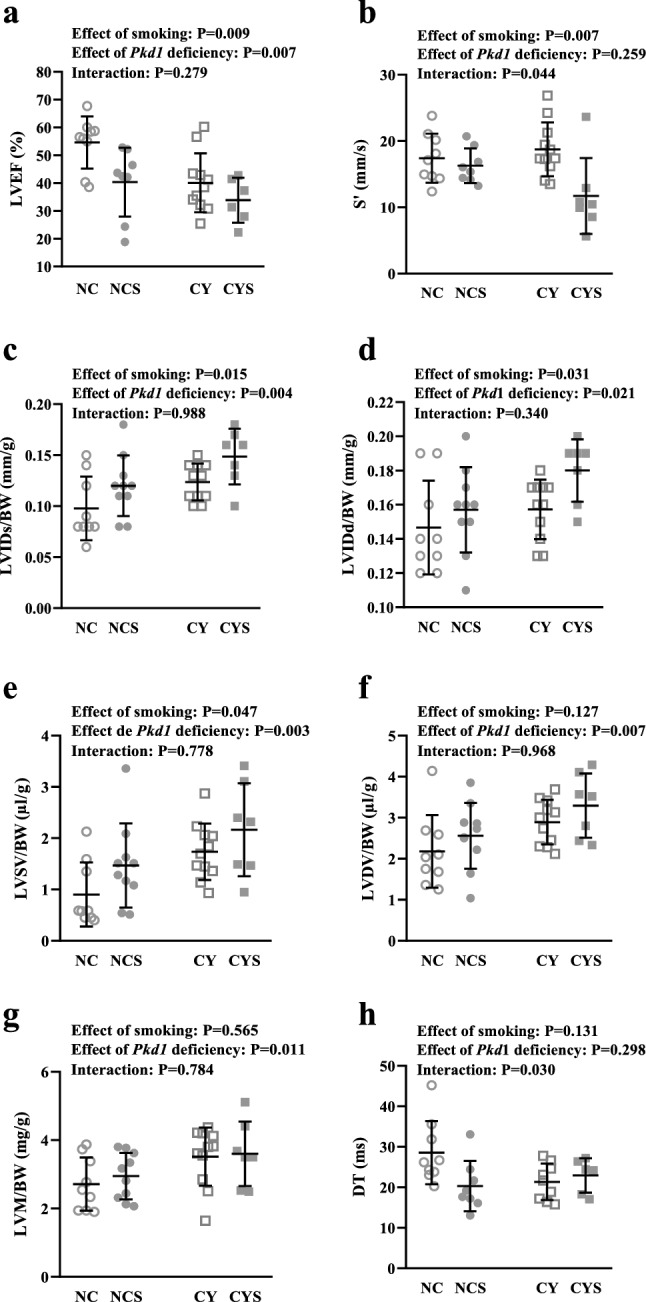
Analyses of the effects of smoking and *Pkd1* deficiency in LVEF (**a**), S’ (**b**), LVIDs/BW (**c**), LVIDd/BW (**d**), LVSV/BW (**e**), LVDV/BW (**f**), LVM/BW (**g**) and DT (**h**) on NC, NCS, CY and CYS mice. These data were analyzed using two-way analysis of variance.

Echocardiographic assessment was also performed at 16 weeks. Our results revealed independent and additive effects of smoking (P = 0.009) and the *Pkd1*-deficient phenotype (P = 0.007) on diminishing the left ventricular ejection fraction (LVEF) measured with the Simpson method (54.59 ± 9.35% in NC, 40.35 ± 12.36% in NCS, 40.07 ± 10.59% in CY, and 33.84 ± 8.08% in CYS; Fig. 3a, Table 1). Independent effects were also shown for reduction of left ventricular shortening fraction (LVSF) (P < 0.001 for smoking and P = 0.005 for *Pkd1* deficiency; Table 1). Notably, systolic velocity (S’) dramatically drops in CYS compared to CY mice but virtually does not change in NCS compared to NC animals, revealing a significant effect of smoking (P = 0.007) with positive interaction with *Pkd1* deficiency (P = 0.044; Fig. 3b, Table 1).

**Table 1:** Echocardiographic parameters in NC, NCS, CY and CYS mice.

Parameters	NC	NCS	CY	CYS	CYST	SMOKING	INTERACTION
			**Structure**		**P**	**P**	**P**
LVM/BW (mg/g)	2.71 ± 0.78 n = 9	2.94 ± 0.68 n = 10	3.51 ± 0.85 n = 11	3.60 ± 0.94 n = 7	0.011	0.565	0.784
LA/BW (mm/g)	0.08 ± 0.02 n = 9	0.08 ± 0.01 n = 10	0.07 ± 0.02 n = 11	0.08 ± 0.01 n = 7	0.264	0.053	0.147
LVIDs/BW (mm/g)	0.10 ± 0.03 n = 9	0.12 ± 0.03 n = 10	0.12 ± 0.02 n = 11	0.15 ± 0.03 n = 7	0.004	0.015	0.988
LVIDd/BW (mm/g)	0.15 ± 0.03 n = 9	0.15 ± 0.02 n = 10	0.16 ± 0.02 n = 11	0.18 ± 0.02 n = 7	0.021	0.031	0.340
LVSV/BW (μl/g)	0.90 ± 0.63 n = 9	1.47 ± 0.82 n = 10	1.74 ± 0.55 n = 11	2.17 ± 0.91 n = 7	0.003	0.047	0.778
LVDV/BW (μl/g)	2.18 ± 0.89 n = 9	2.56 ± 0.80 n = 10	2.89 ± 0.54 n = 11	3.29 ± 0.78 n = 7	0.007	0.127	0.968
IVSD (mm)	0.61 ± 0.08 n = 9	0.69 ± 0.08 n = 10	0.72 ± 0.18 n = 11	0.70 ± 0.10 n = 7	0.146	0.430	0.239
LVPWD (mm)	0.64 ± 0.09 n = 9	0.73 ± 0.10 n = 10	0.70 ± 0.14 n = 11	0.68 ± 0.08 n = 7	0.886	0.395	0.127
			**Systolic function**				
LVEF (%)	54.59 ± 9.35 n = 9	40.35 ± 12.36 n = 8	40.07 ± 10.59 n = 11	33.84 ± 8.08 n = 6	0.007	0.009	0.279
LVSF (%)	65.11 (31.29–69.67) n = 9	22.82 (19.08–30.28) n = 10	32.71 (17.28–36.34) n = 11	16.51 (9.48–23.84) n = 7	0.005	< 0.001	0.051
S´ (mm/s)	17.41 ± 3.71 n = 9	16.28 ± 2.61 n = 8	18.75 ± 4.06 n = 11	11.71 ± 5.73 n = 7	0.259	0.007	0.044
			**Diastolic function**				
IVRT (ms)	26.29 ± 7.90 n = 9	24.77 ± 3.67 n = 9	26.18 ± 4.91 n = 10	34.16 ± 14.94 n = 7	0.113	0.266	0.105
DT (ms)	28.56 ± 7.78 n = 9	20.30 ± 6.21 n = 8	21.36 ± 4.50 n = 9	22.94 ± 4.28 n = 6	0.298	0.131	0.030
			**Other parameters**				
MPI	0.66 ± 0.18 n = 8	0.72 ± 0.15 n = 9	0.67 ± 0.18 n = 11	0.81 ± 0.22 n = 6	0.433	0.126	0.533
RVMPI	0.43 ± 0.23 n = 9	0.49 ± 0.29 n = 7	0.45 ± 0.18 n = 10	0.46 ± 0.16 n = 5	0.976	0.665	0.765
VTI _RVOT_	25.29 (12.65–26.95) n = 9	22.44 (20.57–24.36) n = 10	19.13 (13.31–23.79) n = 11	16.40 (14.66–26.95) n = 6	0.269	0.642	0.627
PAP (mmHg)	66.92 ± 3.10 n = 9	70.17 ± 3.06 n = 9	68.55 ± 5.79 n = 11	69.18 ± 3.03 n = 5	0.831	0.205	0.387

*LVM/BW* left ventricular mass adjusted to body weight, *LA/BW* left atrium diameter adjusted to body weight, *LVIDs/BW)* left ventricular internal diameter in systole adjusted to body weight, *LVIDd/BW* left ventricular internal diameter in diastole adjusted to body weight, *LVSV/BW* left ventricular volume in systole adjusted to body weight, *LVDV/BW* left ventricular volume in diastole adjusted to body weight, *IVSD* intraventricular septum diameter, *LVPWD* left ventricular posterior wall diameter*, LVEF* left ventricular ejection fraction. *LVSF* left ventricular shortening fraction, *S’* peak velocity at the septal basal level, *IVRT* isovolumetric relaxation time, *DT* mitral valve deceleration time, *MPI* myocardial performance index, *RVMPI* right ventricular myocardial performance index, *VTI *_*RVOT*_ velocity–time integral of the right ventricular outflow tract, and *PAP* mean pulmonary artery pressure. Two-way analyses of variance were performed for parametric data and aligned rank transform followed by two-way analysis of variance were performed for non-parametric data.

Structural analyses also revealed independent and additive effects of smoking and *Pkd1* deficiency on increasing the left ventricular internal diameter in systole adjusted to BW (LVIDs/BW) (P = 0.015 and P = 0.004, respectively) and in diastole (LVIDd/BW) (P = 0.031 and P = 0.021, respectively) (0.15 ± 0.03 mm/g and 0.10 ± 0.03 mm/g in NC, 0.15 ± 0.02 mm/g and 0.12 ± 0.03 mm/g in NCS, 0.16 ± 0.02 mm/g and 0.12 ± 0.02 mm/g in CY, and 0.18 ± 0.02 mm/g and 0.15 ± 0.03 mm/g in CYS, respectively; Fig. 3c,d, Table 1). Along this line, smoking and *Pkd1* deficiency independently increased left ventricular volume in systole adjusted to BW (LVSV/BW) (P = 0.047 and P = 0.003, respectively) while the cystic status was associated with a higher left ventricular volume in diastole adjusted to BW (LVDV/BW) (P = 0.007) and a higher left ventricular mass adjusted to BW (LVM/BW) (P = 0.011) (Fig. 3e–g, Table 1). Our data also showed a trend of an independent effect of smoking on increasing the left atrium diameter adjusted to BW (LA/BW) (Table1). No significant effects were detected on intraventricular septum diameter (IVSD) or left ventricular posterior wall diameter (LVPWD).

Analysis of diastolic parameters did not reveal significant effects of smoking and *Pkd1* deficiency, although these conditions interacted with respect to the deceleration time of mitral E wave (DT) (P = 0.030), decreasing in NCS compared to NC and mildly increasing in CYS compared to CY (Fig. 3h, Table 1). Isovolumetric relaxation time (IVRT) was not affected by either factor.

No significant effects of smoking or *Pkd1* deficiency were observed in myocardial performance index (MPI), right ventricular myocardial performance index (RVMPI), mean pulmonary artery pressure (PAP) or velocity–time integral of right ventricular outflow tract length (VTI _RVOT_) (Table 1).

### Smoking increases proliferation of cyst-lining and tubular cells

**Figure 4:**
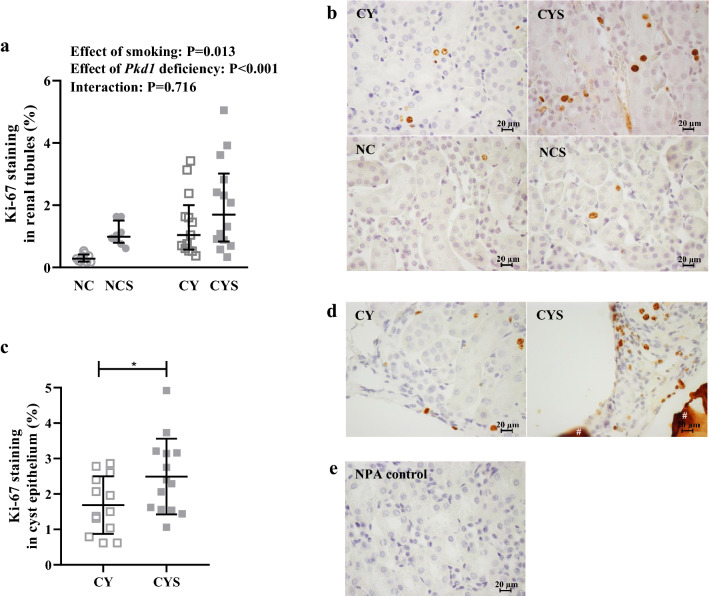
(**a**) Analysis of the effects of smoking and *Pkd1* deficiency in tubular epithelial cell proliferation on NC (n = 9), NCS (n = 8), CY (n = 13) and CYS (n = 14) mice. This analysis was carried out using aligned rank transform followed by two-way analysis of variance. (**c**) Comparative analysis of cell proliferation in renal cystic epithelium between CY (n = 13) and CYS (n = 14) animals. This comparison was performed using unpaired t-test. *P < 0.05. (**b**,**d**) Representative images of Ki-67 staining in renal tubular epithelium and cystic epithelium, respectively. ^#^ Extracellular protein aggregate non-specifically stained. (**e**) Negative control without primary antibody (NPA control) corresponding to the Ki-67 staining images shown in b and d. (**b**,**d**,**e**): Original magnification, × 400; bar = 20 µm.

Ki-67-based assays showed that both the *Pkd1*-deficient cystic phenotype and smoking independently promoted increased proliferation of tubular epithelial cells (P < 0.001 and P = 0.013, respectively), effects that were shown to be additive [0.28% (0.19–0.42) in NC, 0.98% (0.79–1.50) in NCS, 1.04% (0.57–2.01) in CY, and 1.70% (0.83–3.02) in CYS; Fig. 4a,b]. In line with this observation, the proliferation rate of cyst-lining cells was significantly higher in CYS than CY mice (2.49 ± 1.07% *versus* 1.69 ± 0.81%; P = 0.038; Fig. 4c,d; negative control in 4e).

**Figure 5:**
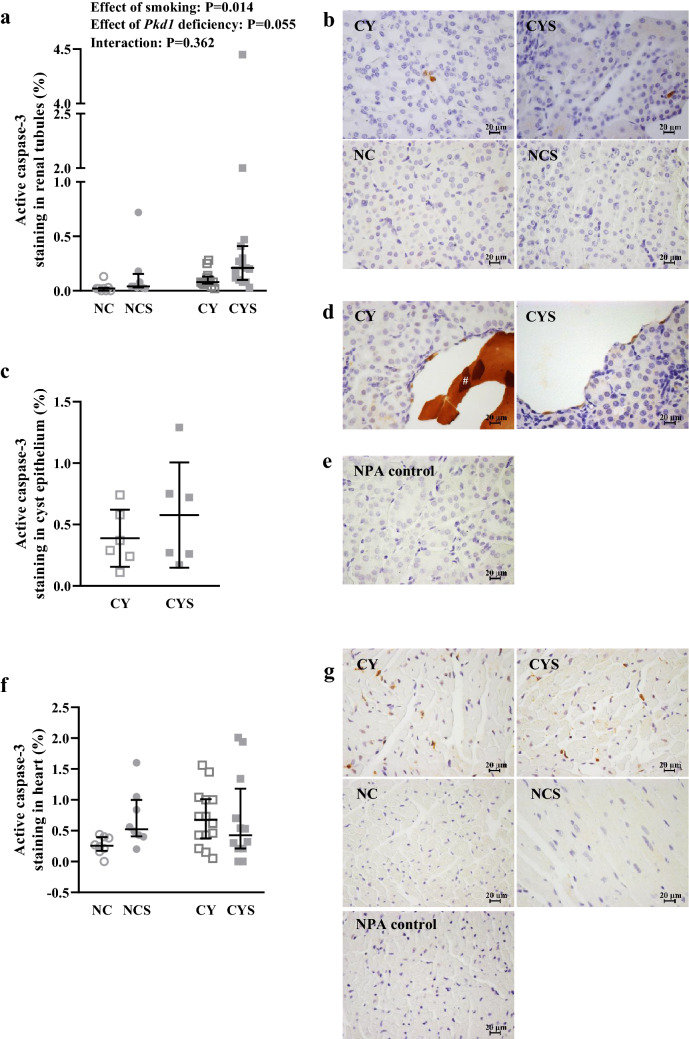
**(a**) Analysis of the effects of smoking and *Pkd1* deficiency in tubular epithelial cell apoptosis on NC (n = 8), NCS (n = 13), CY (n = 8) and CYS (n = 15) mice. This analysis was performed by aligned rank transform followed by two-way analysis of variance. (**c**) Comparative analysis of apoptotic rate in renal cystic epithelium between CY (n = 6) and CYS (n = 6) animals (not significant). This comparison was carried out using unpaired t-test. (**b**,**d**) Representative images of active caspase-3 staining in renal tubular epithelium and cystic epithelium, respectively. ^#^ Extracellular protein aggregate non-specifically stained. (**e**) Negative control without primary antibody (NPA control) corresponding to the active caspase-3 staining images shown in **b** and **d**. (**f**) Analysis of the effects of smoking and *Pkd1* deficiency in the apoptotic rate of cardiac tissue on NC (n = 8), NCS (n = 8), CY (n = 14) and CYS (n = 12) mice. This analysis was performed using aligned rank transform followed by two-way analysis of variance. g) Representative images of active caspase-3 staining in cardiac tissue (b,d,e,g): Original magnification, × 400; bar = 20 µm.

Analyses of active caspase-3 staining profiles revealed that smoking enhanced the apoptotic rate in tubular epithelial cells (P = 0.014), while a trend of a similar effect was detected for the cystic phenotype (P = 0.055) [0.02% (0.00–0.03) in NC, 0.08% (0.07–0.13) in NCS, 0.04% (0.03–0.16) in CY, and 0.21% (0.10–0.41) in CYS; Fig. 5a,b]. The degree of apoptosis in cyst-lining cells, however, was not found to differ between CY and CYS mice (Fig. 5c,d; negative control in 5e).

A separate comparison between CY and NC mice confirmed a previous finding^25^ of increased apoptosis in CY hearts [0.68% (0.38–1.01) *versus* 0.26% (0.17–0.40); P = 0.018]. The 4-group analysis including smoking, however, did not detect independent effects of either *Pkd1* deficiency or smoking on cardiac apoptosis (Fig. 5f,g).

### Renal fibrosis increases with smoking exposure, especially in cystic mice

**Figure 6:**
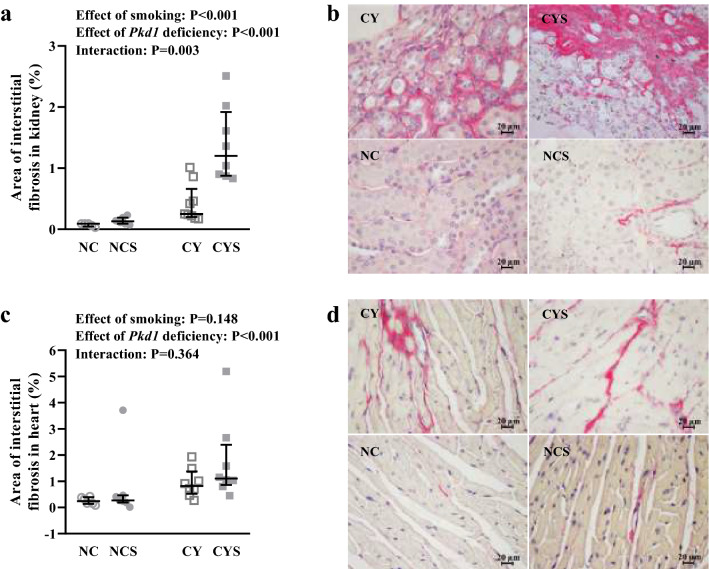
Analyses of the effects of smoking and *Pkd1* deficiency in renal (**a**) and cardiac (**c**) fibrosis index on NC (n = 5), NCS (n = 9), CY (n = 7) and CYS (n = 8) mice. These data were analyzed using aligned rank transform followed by two-way analysis of variance. **b**) Representative images of renal fibrosis and **d**) Representative images of cardiac fibrosis in the four groups. Original magnification × 400; bar = 20 µm.

Tissue fibrosis was quantified based on picrosirius staining, revealing that both smoking and the *Pkd1*-deficient status increase the relative area of fibrosis in the kidneys (P < 0.001 in both cases; Fig. 6a,b). Remarkably, the increase in renal fibrosis promoted by smoking was much higher in cystic than noncystic mice, revealing positive interaction between smoking and *Pkd1* deficiency (P = 0.003) [0.09% (0.04–0.09) in NC, 0.13% (0.09–0.19) in NCS, 0.25% (0.20–0.66) in CY, and 1.20% (0.88–1.92) in CYS].

*Pkd1* deficiency was also associated with increased cardiac fibrosis (P < 0.001), an effect not detected for smoking [0.24% (0.13–0.39) in NC, 0.27% (0.19–0.46) in NCS, 0.81% (0.52–1.37) in CY, and 1.10% (0.86–2.39) in CYS; Fig. 6c,d].

### Renal levels of glutathione are reduced in cystic and noncystic mice submitted to smoking

**Figure 7:**
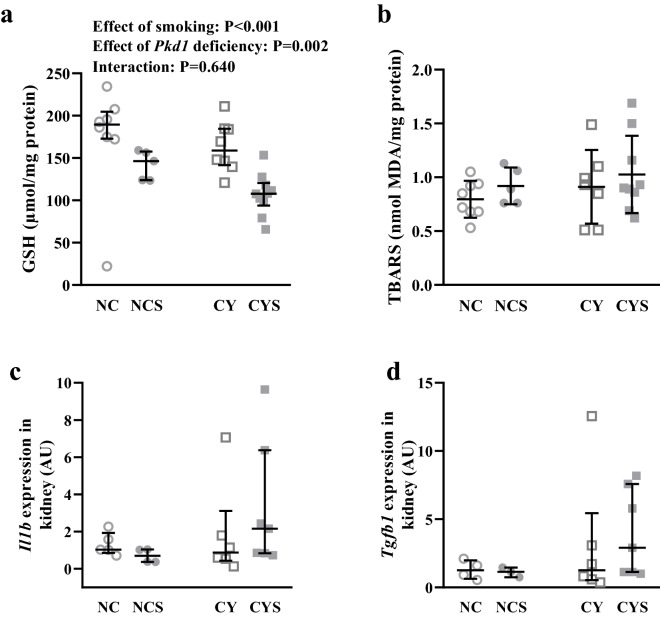
(**a**) Analysis of the effects of smoking and *Pkd1* deficiency in the renal glutathione (GSH) levels on NC (n = 8), NCS (n = 5), CY (n = 8) and CYS (n = 10) mice. (**b**) Analysis of the effects of smoking and *Pkd1* deficiency in the renal thiobarbituric acid reactive substances (TBARS) levels on NC (n = 8), NCS (n = 5), CY (n = 7) and CYS (n = 9) animals. (**c**) Analyses of the effects of smoking and *Pkd1* deficiency in *Il1b* expression in renal tissue on NC (n = 5), NCS (n = 4), CY (n = 6) and CYS (n = 7) mice; and (**d**) in *tgfb1* expression in renal tissue on NC (n = 5), NCS (n = 3), CY (n = 6) and CYS (n = 7) animals. The data were analyzed by aligned rank transform followed by two-way analysis of variance (**a**,**c**,**d**) and by two-way analysis of variance (**b**).

Exposure to smoking and the cystic phenotype independently exerted reducing effects on renal levels of glutathione (GSH) (P < 0.001 and P = 0.002, respectively), effects shown to present an additive behavior [189.46% (174.91–204.65) in NC, 146.29% (123.79–157.62) in NCS, 158.77% (141.46–184.48) in CY, and 107.89% (93.82–120.45) in CYS; Fig. 7a].

Quantitative analyses of thiobarbituric acid reactive substances (TBARS) in renal tissue did not reveal significant effects of smoking and the cystic phenotype (Fig. 7b).

### Smoking did not significantly affect *Il1b* and* Tgfb1* renal expression

The inflammatory status in renal tissue was analyzed by real-time RT-PCR. Expression levels of *Il1b* and *Tgfb1* did not significantly differ among the groups, despite higher median values in CYS mice (Fig. 7c,d).

## Discussion

The identification of environmental risk factors for faster progression of ADPKD carries great medical interest, as the avoidance or cessation of such stimuli is expected to prevent acceleration or slow disease progression. Smoking has been investigated as one of these potential risk factors. Preclinical studies have shown that nicotine increases vasopressin levels^29^, which increases renal cyclic AMP and favors cyst growth^30^. In addition, smoking has been independently associated with renal disease progression in this disorder^10^. Another clinical study revealed that male smokers with ADPKD display increased risk of reaching ESKD^9^. Interestingly, while the risk of progressing to ESKD was substantially higher in smokers with no history of ACE inhibitor (ACEi) use, the odds ratio for smokers submitted to this treatment was not significant. The reasons for this finding remained unclear. Recent reports, however, found that smoking status did not affect renal survival in ADPKD^11^ or that smoking history did not differ between progressor and nonprogressor patients^31^. These data brought controversy to the smoking-ADPKD scenario, in addition to an already existing lack of information on whether the reported tobacco-related effects follow mechanisms common to all forms of CKD or are mainly based on deleterious actions specific to ADPKD. At this point, a robust in vivo experimental study became essential to clarify and understand key points of the mentioned association.

The origin of renal cysts in the *Pkd1*^flox/flox^:*Nestin*^cre^ mouse was investigated in a previous study, which found that renal cysts are derived predominantly from collecting ducts/distal tubules at P28 and P49^32^. In the current study we performed this evaluation at 18 weeks of life (including *Pkd1*^flox/flox^:*Nestin*^cre^ and *Pkd1*^flox/-^:*Nestin*^cre^ mice), analyzing the DBA and LTL staining patterns. Although at lower rates, our findings also revealed a high proportion of cysts derived from collecting ducts/distal tubules. Taken together, the data from the two studies suggest that cysts derived from collecting ducts/distal tubules tend to form/grow earlier than cysts derived from proximal tubules in this animal model. Since cysts derived from collecting ducts tend to be larger and more numerous in human ADPKD^33^, the two reports also support that the *Pkd1*^flox/flox^:*Nestin*^cre^/*Pkd1*^flox/-^:*Nestin*^cre^ mice constitute an appropriate model to investigate different aspects of human ADPKD. An interesting question, however, is how these animals can develop a high proportion of collecting duct-derived renal cysts if nestin is not detected in ureteric bud and its derivatives nor later in collecting ducts. A possible explanation includes three points: (1) collecting duct cells are significantly more prone to originate cysts associated with *PKD1/Pkd1* or *PKD2/Pkd2* deficiency, since in ADPKD the preferential cyst origin is the collecting duct; (2) cyst formation originates from an absolute minority of cells from an absolute minority of nephrons; and (3) in this scenario, even expression of nestin in an extremely low number of collecting duct cells (not enough to be detected in previous studies) along a certain time period could potentially lead to significant cyst development from such cells. Interestingly, our data showed that at an older age our mice also display a significant proportion of cysts derived from proximal tubules. At this point, however, we have no good explanation as to why in these cysts LTL staining is observed in only a fraction of cyst-lining cells.

The smoking protocol employed in the current study was early and long enough to detect potential deleterious effects at all several pathogenic steps, including cystogenesis, cystic growth, fibrosis, hemodynamics and other basic cellular alterations. To ensure this detection capacity, we worked with high exposure to cigarette smoke at doses equivalent to those used in previous studies^34,35^.

Smoking has been shown to affect the kidney in different ways. It can cause albuminuria and/or proteinuria, increase the renal arteriolar wall, and lead to hemodynamic changes associated with hypoperfusion^18,36^. Our results revealed that smoking increased SUN in cystic and noncystic mice, indicating that its deleterious effect on renal function was not specific to the underlying disease. It is possible that a significant component of this effect resulted from hemodynamic alterations induced by sympathetic exacerbation and increase in renal vascular resistance^20^.

Chronic exposure to smoking significantly increased the renal cystic index, revealing a marked deleterious effect on the most seminal phenotype of ADPKD. Data and image analyses indicate that most of this effect occurred due to acceleration of cystic growth. Limitations of the employed methodology did not allow appropriate investigation into whether the smoking-related increase in cystic burden also involves acceleration of cystogenesis. It is possible that somatic mutations induced by tobacco contribute to the formation of new renal cysts, by affecting the previously normal *Pkd1* allele of tubular cells in *Pkd1*^flox/-^:*Nestin*^cre^ mice or the previously normal *Pkd1* allele of *Pkd1*^flox/flox^:*Nestin*^cre^ tubular cells in which the Cre-lox system inactivated only one of the two copies, a phenomenon previously described for other targeted genes^37,38^. Such somatic mutations would have occurred during renal development, behaving as the second hit for cystogenesis. Smoking could have also induced second hits in post-kidney development cells, allowing them to evolve to cystogenesis in the presence of a third hit^39^. Two-hit cells localized in ischemic areas provoked by the hemodynamic effects of smoking could potentially acquire this third hit.

Our data showed that smoking increased cell proliferation of tubular cells. It is particularly relevant to note that smoking also led to higher cell proliferation rates in cyst epithelia. The increase in cystic index in response to smoking suggests that the tobacco proproliferative effect, although not specific to *Pkd1* deficiency, is particularly important at the cyst-lining-cell level, playing an essential role in smoking-induced cyst growth. Based on a study carried out in aortic smooth muscle cells, it is possible that this proproliferative effect be exerted by nicotine^40^.

Smoking increased renal fibrosis in cystic and noncystic mice; this effect, however, was much higher in cystic kidneys. Our results confirm that cystic kidneys present increased levels of renal fibrosis^25^ and suggest that smoking may stimulate renal fibrosis in ADPKD, a process critically associated with disease progression^41^. We did not find, however, increase in *Il1b* and *Tgfb1* expression levels in cystic kidneys following exposure to smoking. It is possible that these results occurred due to high variability among animals, since *Il1b* and *Tgfb1* levels were numerically higher in CYS than CY mice. Interestingly, smoking led to cystic index increase in CYS animals without increase in KW/BW, suggesting parenchymal reduction in CYS kidneys. Increased renal fibrosis may have been a significant contributor to this likely decrease in renal parenchyma.

Redox imbalance is another important component of ADPKD pathogenesis^42^. ADPKD patients present decreased levels of glutathione peroxidase and superoxide dismutase^43^. Additionally, chronic smoking induces oxidative stress and damages endothelial cells^44^, and cigarette substances reduce glutathione levels in mouse lung epithelial cells^17^. We have confirmed in our animals an effect of the cystic phenotype on decreasing renal glutathione levels. As an additive effect, smoking also independently reduced the kidney glutathione content, leading to further diminishment in CYS mice. These data suggest that oxidative stress may participate in the detrimental effect of smoking on *Pkd1*-deficient renal disease, although its induction is not specific to cystic kidneys.

Apoptosis is another pathogenic component in ADPKD^45^. Nicotine, in turn, has been reported to induce or inhibit apoptosis in different cell types^40,46^. Our data revealed that smoking independently increased tubular cell apoptosis, while the cystic phenotype was associated with a similar trend. Interestingly, however, this proapoptotic effect of smoking could not be detected in cyst-lining cells. In this scenario, our results suggest that increased tubular epithelial apoptosis may be a contributing factor to the deleterious effect of smoking on cystic kidneys but do not support apoptosis as a contributor to the effect of smoking on cyst growth.

Recent experimental studies support a fundamental role of gene deficiency, *PKD1* or *PKD2*, in the development of ADPKD-associated cardiomyopathy^7,25,26,47–49^. Other factors, including hypertension, may also contribute to cardiac deterioration in this disorder^50^. Smoking, in turn, is associated with harmful cardiovascular effects^51^. Our data confirmed that the *Pkd1*-deficient status independently impacts systolic function, reducing LVEF and LVSF. They also showed an independent effect of smoking on decreasing LVEF and LVSF. Interestingly, smoking interacted with *Pkd1* deficiency with respect to S’, severely reducing this parameter in cystic mice while a very mild effect was detected in noncystic animals. This observation revealed that diminished *Pkd1* functional activity can sensitize the myocardium for a detrimental effect of smoking, enlarging its magnitude. Diastolic parameters, however, were not significantly impacted by smoking.

Notably, smoking worsened several structural parameters in cystic mice. Smoking and cardiac *Pkd1* deficiency independently and additively increased LVIDs/BW, LVIDd/BW and LVSV/BW. It should be noted that in humans LVIDs and LVIDd are risk factors for congestive heart failure in the absence of a previous myocardial infarction^52^. Altogether, our data indicate that smoking exerts deleterious effects on the hearts of *Pkd1*-deficient cystic mice, including worsening in systolic function and detrimental structural responses. These findings are highly relevant to the cystic scenario, since the parameters associated with these cardiac abnormalities are already impaired in nonsmoking cystic mice due to the underlying cardiomyopathy primarily caused by *Pkd1* deficiency.

Our data confirmed increased cardiac fibrosis in cystic mice^25^. Larger fibrotic areas were detected in *Pkd1*-deficient hearts, whereas smoking did not impair this phenotype. Our findings suggest, in addition, that apoptosis is not involved in the smoking effect upon the heart of cystic animals.

Survival was not significantly different among the groups at 18 weeks of life. A trend towards lower survival in the CYS group compared to NC, however, suggests the possibility that the coexistence of *Pkd1* deficiency and smoking exposure may have a detrimental effect on survival. Interestingly, smoking induced a substantial reduction in body weight in cystic animals, while an almost negligible effect was observed in noncystic mice. This positive interaction between *Pkd1* deficiency and smoking expands the concept that ADPKD is a systemic disease. Future studies addressing this issue should measure caloric intake and metabolic rate to distinguish between primary and secondary effects of *Pkd1* deficiency on body weight.

Our findings showed that chronic exposure to smoking had a deleterious effect on the renal phenotype of *Pkd1*-deficient cystic mice, accelerating cystic growth and renal fibrosis as well as reducing renal function. Our results suggest that increased cell proliferation contributed to cyst growth and show that smoking reduced the renal levels of glutathione. Our data also revealed detrimental consequences of smoking on the cardiac phenotype of cystic mice, both at the functional and structural levels. Given the similarities between human ADPKD and the studied orthologous animal model, the observed deleterious effects of smoking are likely to apply to ADPKD patients.

## Methods

### Ethical approval

This project was approved by the Research Ethics Committee of the University of São Paulo School of Medicine, São Paulo, Brazil, under the approval number 098/2015. This study followed the national and international standards for animal care and experimentation and is reported in compliance with the ARRIVE guidelines.

### Mouse model

Cystic mice were generated based on a *Pkd1* floxed allele (*Pkd1*^flox^) with loxP sites positioned in introns 1 and 4 and a neomycin cassette flanked by FRT sites located in intron 1^53^. The presence of this allele was detected in the animal by genotyping with the primers 5’-CCTGCCTTGCTCTACTTTCC-3’ (F4) and 5’-AGGGCTTTTCTTGCTGGTCT-3’ (R5), a PCR protocol that yields a 250-bp product. Excision of exons 2–4 was induced by expression of Cre recombinase under the control of the Nestin promoter. The recognition of this transgene was also performed by a PCR reaction, in this case using the primers 5’-ATTGCTGTCACTTGGTCGTGGC-3' (CreF) and 5’-GGAAAATGCTTCTGTCCGTTTGC-3’ (CreR), which produces a product of 200 bp. *Pkd1*^flox/flox^ were distinguished from *Pkd1*^flox/-^ mice by a genotyping procedure targeting the *Pkd1* inactivated allele, using the primers F4 and 5’-TCGTGTTCCCTTACCAACCCTC-3’ (R4). The presence of a 200-bp product identified *Pkd1*^flox/-^ animals while the lack of it indicated the *Pkd1*^flox/flox^ genotype. *Pkd1*^flox/flox^:*Nestin*^cre^ mice, in turn, were distinguished from *Pkd1*^flox/-^:*Nestin*^cre^ animals by SYBR Green-based qPCR (PowerUp SYBR Green Master Mix, Thermo Fisher, Massachusetts, EUA) including parallel amplifications of the F4-R5 product (*Pkd1*^flox^ allele) and the F4-R4 product (*Pkd1*^-^ allele). Similar or slightly higher amplification of F4-R4 compared to F4-R5 identified *Pkd1*^flox/-^:*Nestin*^cre^ mice, while a much higher amplification of F4-R5 with respect to F4-R4 defined the animals as *Pkd1*^flox/flox^:*Nestin*^cre^.

The mice were generated and maintained on a C57BL/6 background. The cystic *Pkd1*^flox/flox^:*Nestin*^cre^ and *Pkd1*^flox/-^:*Nestin*^cre^ animals display a mild to moderate renal cystic phenotype that resembles human ADPKD. *Pkd1*^flox/flox^ noncystic animals were employed as controls.

All experiments were performed in a single gender, male mice, to avoid potential gender-related experimental heterogeneity. This study was carried out in accordance with international standards of animal care and experimentation.

### Exposure to cigarette smoke

Female *Pkd1*^flox/flox^ noncystic mice of reproductive age were exposed to smoking and mated with *Pkd1*^flox/flox^:*Nestin*^cre^ or *Pkd1*^flox/-^:*Nestin*^cre^ cystic males not exposed to it. The mating system was temporary and polygamous. Once copulation was confirmed, pregnant females were isolated in individual cages. Tobacco exposure was extended throughout the gestational period and the postnatal trial. CY and NC controls were not submitted to pre- or post-natal exposure to smoking.

The smoking protocol was carried out using a previously described system^54^ and comprised two 30-min periods/day, with a minimum interval of 4 h, five times/week. Seven to nine cigarettes (Derby red pack, Souza Cruz, Rio de Janeiro, Brazil) were burned per period, following a priorly established protocol^34,35^. Smoke exposure was carried out within a plastic box with three openings: one at the top, for air and smoke exit; and two at the sides, one for synthetic air intake and another for cigarette smoke entry. Smoke was drawn into the box by a Venturi system connected to a lit cigarette. Carbon monoxide (CO) concentration was monitored inside the smoking box with a CO sensor (Toxi-Oxy, Biosystems, Connecticut) and maintained at 250–350 ppm, a range associated with non-toxic carboxyhemoglobin levels^55^.

### Biochemical determination of serum urea nitrogen

Blood samples were collected by retroocular access in anesthetized animals. SUN was determined using an enzymatic-colorimetric method (Urea CE kit, Labtest, MG, Brazil).

### Renal ultrasonographic evaluation

Renal ultrasonographic analysis was performed in CYS and CY mice using the Vevo 2100 equipment (VisualSonics Inc., Toronto, Canada) with a 40-mHz transducer. The animals were anesthetized with isoflurane and two-dimensional images were acquired for each kidney. The three renal axes were measured and an axial-cut film of 3 s and 200 images was generated. Renal volume was calculated using the ellipsoid volume modified equation^56^ while cyst volume was obtained based on its diameter and the assumption of a spherical structure. The renal cystic index was the ratio between the sum of the cyst volumes of both kidneys and the total volume of both organs.

### Echocardiographic analyses

Echocardiographic assessment was performed with the same device and basic procedures used for renal ultrasonography but with the animal in dorsal decubitus. Image acquisition in one-dimensional mode (M-mode) was used for analysis of LVIDd and LVIDs, for the calculations of LVSF and LVM, and to obtain LA. Two-dimensional mode (B-mode) was used for the analysis of LVDV and LVSV, measures used in the calculation of LVEF using the Simpson method^57^. Analyses of DT and PAP were performed with pulsatile Doppler (PD)^58^. Tissue Doppler (TD) imaging, in turn, allowed the acquisition of S’, IVRT and MPI. VTI _RVOT_ and RVMPI were also acquired with TD.

### Organ harvesting and tissue histological preparation

Eighteen-week-old mice were anesthetized with intraperitoneal thiopental (0.4 mg/g of BW) and submitted to thoracotomy, insertion of a catheter into the left ventricle and a cut in the right atrium for blood drainage. Saline solution was infused until complete exsanguination, followed by immediate harvesting and weighing of kidney, heart and liver. The right kidney and part of the heart were fixed in 4% formaldehyde for 24 h, processed in a graded series of ethanol, embedded in paraffin, subjected to 3-μm-thick sections, and submitted to hematoxylin and eosin and Sirius red staining.

### Immunohistochemical evaluation of cell proliferation, apoptosis and cyst origin

Cell proliferation was analyzed using an anti-Ki-67 monoclonal antibody (ab16667, ABCAM, Cambridge, UK). Negative control reactions had the primary antibody replaced with the diluent while positive control ones were performed in mouse spleen sections. Each kidney section had eight cortical and two medullary fields analyzed at 400 × magnification. Cell proliferation rate was calculated as the ratio cells with positive nuclei/total cells.

Apoptosis was evaluated with an anti-active caspase-3 antibody (CP229B, BioCare Medical, Concord, CA). Control reactions and selection of kidney fields followed the same protocol used for Ki-67. Each heart section had 10 random fields analyzed. Quantification was performed as the ratio positive cells/total cells.

Cyst origin was analyzed using the biotinylated lectins *Dolichos biflorus* agglutinin (DBA) and *Lotus Tetragonolobus* (LTL) (Vector Laboratories, Burlingame, CA; cat.# B-1035 and B-1325, respectively). Cysts were defined as having a diameter ≥ 50 μm^59^. To better characterize the patterns, we classified the cysts as small (diameter ≥ 50 μm and ≤ 300 μm) or large (diameter > 300 μm). Quantification was carried out by counting the number of positive and negative large and small cysts at a 200 × magnification. The results were expressed in percentage, as the ratio number of positive cysts/total number of cysts.

### Tissue fibrosis quantification

Kidney and heart sections were stained with 0.2% picrosirius (Sirius Red, Direct Red 80, Milwaukee, WI). Positive staining was analyzed and quantified using an optical polarizer (BX-51, Olympus, Hatagaya, Japan) with a light polarizer. Each kidney section had 12 cortical and three medullary fields scanned whereas 10 random fields were analyzed for each cardiac tissue slide, at 400 × magnification. Image ProPlus 6.0 software (Media Cybernetics Inc., Rockville, MD) was used for image processing, allowing selection of reddish, orange or greenish birefringent shades for collagen quantification. Data were expressed as the ratio collagen content area/total area.

### Quantification of *Il1b* and *Tgfb1* expression by real-time RT-PCR

Total RNA was extracted from renal tissue using Trizol (Invitrogen, Carlsbad, CA). RNA samples were treated with DNAse (RQ1 RNAse free DNAse, Promega, Fitchburg, WI) and reverse transcription was performed with the ImProm-II kit (Promega, Fitchburg, WI). Real-time PCR reactions were carried out using the StepOne Plus equipment (Thermo-Fisher) and *TaqMan* assay (Applied Biosystems, Warrington, UK) for the specific assays *Il1b* (Mm00434228_m1) and *Tgfb1* (Mm01178820_m1), as well as for the endogenous control *Gapdh* (Mm99999915_g1). Products of qPCR reactions were quantified using the relative ΔΔCt method.

### Analysis of redox metabolism

GSH concentration was quantified in renal tissue using the Glutathione Fluorescent Detection kit (Arbor Assays, MI) and normalized for protein content according to the manufacturer's instructions. Concentration of TBARS was also determined in kidney tissue, utilizing the OxiSelect TBARS assay kit (Cell Biolabs, San Diego, CA), and normalized to protein content.

### Statistical analyses

The data were classified in parametric or nonparametric based on the histogram analysis and the Shapiro-Wilks test. Parametric data are expressed as mean ± standard deviation (SD) and nonparametric data as median and interquartile range. For parametric data, comparisons between two groups were performed using the t-test with or without Welch’s correction, depending on the analysis of equality of variances carried out with the Levene test. Two-factor comparisons for parametric data were performed with two-way analysis of variance employing the type-III sum of squares. When interaction between factors was detected, their interaction was analyzed and interpreted based on the means and graphical analysis of the dot plots.

Comparisons of nonparametric data including two groups were performed employing the Mann–Whitney test. Two-factor analyses for nonparametric data, in turn, were carried out using a robust rank-based analysis of variance, known as aligned rank transform^60^. When interaction between factors was detected, analysis of their interaction was interpreted based on the medians and the dot plots. Survival data were plotted in the Kaplan–Meier curve and compared with the logrank test. We accepted α risk ≤ 5% and β risk ≤ 20%.

To avoid potential statistical bias, *Pkd1*^flox/flox^:*Nestin*^cre^ and *Pkd1*^flox/-^:*Nestin*^cre^ mice were balanced across the factor “smoking” allowing a frequency difference below 20% between the CY and CYS groups. In addition, when significance was detected in any statistical test, we also calculated whether a proper balance between the groups was indeed achieved for the specific variable. This analysis was initiated by calculating the highest numerical value in the 95% confidence interval (CI) of the difference between *Pkd1*^flox/flox^:*Nestin*^cre^ and *Pkd1*^flox/-^:*Nestin*^cre^ for the specific variable. Next, we applied this value and the frequency differences of *Pkd1*^flox/flox^:*Nestin*^cre^ and *Pkd1*^flox/-^:*Nestin*^cre^ animals between the CYS and CY groups to calculate the maximum percent effect on the mean/median potentially caused by the difference in genotype frequency within the 95% CI. Although an effect < 0.2 is accepted as small, in our analyses we adopted the more stringent threshold of 0.1 to guarantee appropriate group balance. Such a low cutoff, associated with the very high probability that the mentioned effect is lower than the highest value within the 95% CI, granted a remarkable reduction in potential bias introduced by the presence of the two genotypes in the CY and CYS groups. When a maximum 95% CI effect > 0.1 was detected, we used the *Pkd1*^flox/flox^:*Nestin*^cre^ or *Pkd1*^flox/-^:*Nestin*^cre^ genotypes as a fixed factor in the multifactorial analysis.

Statistical programs included SPSS 25 (IBM, Armonk, NY), GraphPad 8.0 (Prism Software, GraphPad Software, La Jolla, CA) and R 4.0 (R Foundation for Statistical Computing, Vienna, Austria).

## Supplementary Information

Supplementary Information.

## Data Availability

All results analyzed in this study are included in this article. More information can be requested from the corresponding author via e-mail.
