# Supplementary material for: Smoking accelerates renal cystic disease and worsens cardiac phenotype in Pkd1-deficient mice
Source: Sci Rep. 2021 Jul 14;11:14443. doi: 10.1038/s41598-021-93633-7 (PMC8280209; doi:10.1038/s41598-021-93633-7)
Supplement: Supplementary file 1 — Supplementary Information. [file 41598_2021_93633_MOESM1_ESM.pdf]

Smoking accelerates renal cystic disease and worsens cardiac phenotype in *Pkd1*-deficient mice

Marciana V. Sousa[1], Andressa G. Amaral[1], Jessica A. Freitas[1], Gilson M. Murata[1], Elieser H. Watanabe[1], Bruno E. Balbo[1], Marcelo D. Tavares[2], Renato A. Hortegal[2], Camila Rocon[2], Leandro E. Souza[2], Maria C. Irigoyen[2], Vera M. Salemi[2], and Luiz F. Onuchic[1,*].

[1]Divisions of Nephrology and Molecular Medicine, Department of Medicine, University of São Paulo School of Medicine, São Paulo, Brazil; [2]Heart Institute, University of São Paulo School of Medicine, São Paulo, Brazil.

Supplementary Figure S1

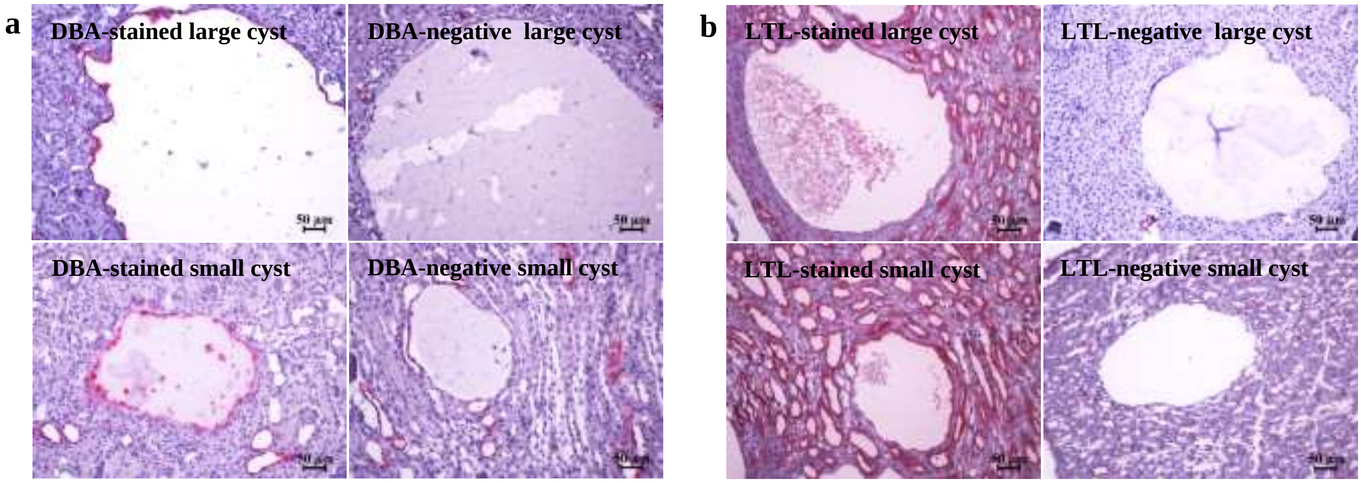

SUPPLEMENTARY FIGURE LEGENDS

Supplementary Figure S1. Representative images of DBA-stained and LTL-stained large and small renal cysts, and of corresponding non-stained cysts. Original magnification x200; bar=50μm.
